# Estimating the budget impact of orphan medicines in Europe: 2010 - 2020

**DOI:** 10.1186/1750-1172-6-62

**Published:** 2011-09-27

**Authors:** Carina Schey, Tsveta Milanova, Adam Hutchings

**Affiliations:** 1 GMAS, Building 3, Chiswick Business Park, London, W4 5YA, UK; 2 Celgene Corporation, 86 Morris Avenue, Summit, NJ 07901, USA

## Abstract

**Background:**

Orphan drugs are a growing issue of importance to European healthcare policy makers. The success of orphan drug legislation in Europe has resulted in an increasing number of licensed medicines for rare diseases, and many more yet unlicensed products have received orphan drug designation. Increasingly the concerns amongst policy makers relate to issues of patient access and affordability, yet few studies have sought to estimate the future budget impact of orphan drugs. The aim of this study was to predict the total cost of orphan medicines in Europe between 2010 and 2020 as a percentage of total European pharmaceutical expenditure.

**Methods:**

A disease-based epidemiological model was created based upon trends in the designation and approval of new orphan medicines, prevalence estimates for orphan diseases, and historical price and sales data for orphan drugs in Europe (defined as Eurozone + UK). The analysis incorporated two stages:

1) Predicting the number of diseases for which new orphan drugs will be approved over the next decade, based on an analysis of trends from the EU registry of orphan medicines;

2) Estimating the average ex-factory drug cost across an orphan disease life cycle, from the year in which the first orphan medicine is launched to the point where the first medicine loses marketing exclusivity.

The two sets of information were combined to quantify the annual cost of orphan drugs from 2010 through 2020.

**Results:**

The results from the model predicted a steady increase in the cumulative number of diseases for which an orphan drug is approved, averaging just over 5 new diseases per year over the next 10 years. The annual per patient cost of existing orphan drugs was seen to vary between €1,251 and €407,631, with the median cost being €32,242 per year. The share of the total pharmaceutical market represented by orphan drugs is predicted to increase from 3.3% in 2010 to a peak of 4.6% in 2016 after which it is expected to level off through 2020, as growth falls into line with that in the wider pharmaceutical market. In sensitivity analysis peak-year orphan drug budget impact ranged between 3% - 6.6%.

**Conclusions:**

Although European orphan drug legislation has led to an increase in the number of approved orphan drugs, the growth in cost, as a proportion of total pharmaceutical expenditure, is likely to plateau over the next decade as orphan growth rates converge on those in the broader pharmaceutical market. Given the assumptions and simplifications inherent in such a projection, there is uncertainty around the base case forecast and further research is needed to monitor how trends develop. However, fears that growth in orphan drug expenditure will lead to unsustainable cost escalation do not appear to be justified. Furthermore, based on the results of this budget impact forecast, the European orphan drug legislation is not leading to a disproportionate impact on pharmaceutical expenditure.

## Background

Orphan diseases are rare and often debilitating conditions, defined in the European Union (EU) as having a prevalence of no more than five per 10,000 people [1]. There are believed to be between 5,000 and 8,000 different rare diseases affecting an estimated 29 million people in the EU [2]. Orphan drugs are those medicines used in the diagnosis, prevention or treatment of orphan diseases.

European legislation passed in 2000 explicitly recognized the unmet need of targeted treatments for orphan diseases and created regulatory pathways and incentives for manufacturers to develop orphan drugs [3]. This regulation is widely perceived to have been a success. From April 2000, when the EU orphan drug regulation came into effect, until October 2010, 720 drugs had received orphan drug designation from the European Medicines Agency (EMA) [4]. Of those designated drugs, 63 had been granted Marketing Authorisation (MA), although some drugs had obtained orphan drug designation and MA for more than one indication (for example, imatinib received its initial MA for chronic myeloid leukemia in 2001, and then further approvals for additional orphan indications at later time points).

While orphan drug designation and MA are EU centralised procedures, decisions on pricing, reimbursement and funding for orphan medicines remain the responsibilities of Member States. This has led to uneven access to orphan medicines across Europe [5]. Increasingly, issues of orphan drug funding and access to medicines are becoming the focus of discussion amongst health policy makers at a European, country and local level. Concerns about the cost of orphan medicines, at a per-patient level and in aggregate, are delaying the acceptance and uptake of these medicines [6]. Policy makers and healthcare managers are concerned that, if left unmonitored, the future growth in orphan drug cost will be prohibitive, and some have questioned whether the orphan drug regulation needs to be revised [7,8].

Yet there is little published evidence upon which to assess the current or future budget impact of orphan medicines in Europe. Those studies that have attempted to estimate budget impact are described below in chronological order by the year of the budget impact estimate.

An analysis conducted in 2004 on behalf of the European Commission predicted the future budget impact of orphan drugs based upon the uptake of products that had been introduced since the legislation was enacted in 2000 [9]. The researchers mapped the prices and accessibility of orphan drugs in Europe, using a range of data sources. Results showed that orphan drugs (those with orphan drug designation) accounted for between 0.7 and 1.0% of pharmaceutical expenditure in 2004 in France and Netherlands, and predicted this would rise to between 6 - 8% by 2010. It is noteworthy that this study was performed in 2004, when limited knowledge on the trends of orphan drugs in Europe was available.

A research thesis compiled by a Dutch student used data from published literature and interviews to investigate the cost of orphan drugs in the Netherlands. The study estimated that the budget impact for orphan drugs reimbursed for use outside of hospital ('extramural') accounted for 0.48% of the total Dutch drug expenditure in 2006 [10]. The author stated that extramural orphan drugs represented 68% of all orphan medicines expenditure in the Netherlands, suggesting that the total budget impact of orphan medicines would have been approximately 0.75% of total pharmaceutical spend in 2006.

Orofino et al [11] performed a retrospective, cross-sectional study on 38 orphan drugs in France, Germany, Italy, Spain and the UK to determine the overall cost of orphan drugs per country, compared to the total drugs spend in 2007. Published disease prevelance data was combined with IMS Health drug prices and manufacturer information on recommended annual dosage for all licensed orphan medicines. The authors found that the average budget impact of orphan medicines accounted for 1.7% of the total drugs expenditure across the five countries.

An analysis conducted by the Belgian Federal Centre for Healthcare (KCE) [12] calculated the budget impact of orphan drugs as a percentage of the total Belgian drug budget in 2008, and then forecast the impact through 2013. By using three different scenarios reflecting different levels of growth in orphan drugs in the EU, the number of drugs reimbursed in Belgium, and the average annual cost per patient, the authors estimated that orphan medicines accounted for 1.9% of total pharmaceutical expenditure in 2008, and predicted it to grow to approximately 4% in 2013.

The most recent estimate of orphan drug budget impact comes from Germany, where according to the 'Arzneiverordnungsreport' (Prescription drugs report), orphan drugs accounted for 2.5% of Statutory Health Insurance spending on drugs in 2009 [13].

The aim of this study was to estimate the European budget impact of orphan medicines as a percentage of total pharmaceutical expenditure, between 2010 and 2020, based upon 10 years of orphan drug experience in Europe.

## Methods

For the purposes of this study Europe was defined as the Eurozone countries (AT, BE, CY, DE, EE, ES, FI, FR, GR, IE, IT, LU, MT, NE, PT, SK, SL) plus the UK. These markets were considered to be more homogenous in their approach to the provision of orphan medicines than the broader EU-27 countries, where large disparities in access to orphan medicines have been observed [5].

The analysis only incorporated the cost associated with medicines that have orphan drug designation. It did not account for drugs that are used to treat rare diseases but which were licensed prior to the introduction of the new legislation in 2000, nor those for which the manufacturers did not seek orphan drug status. This approach is in accordance with the previous studies in this area and reflects the primary concern of healthcare providers, which is the cost of new medicines launched on the back of the 2000 orphan drug legislation [11,12].

A disease-based epidemiological modeling approach was utilised to predict the future budget impact of orphan drugs based upon historical trends and epidemiological data. The primary unit of measurement in the model is the orphan disease, rather than the orphan drug. For any given year in the model, the total cost of orphan drugs is estimated according to the number of orphan diseases multiplied by the average orphan disease medicine cost.

### Predicting the number of orphan diseases

For costing purposes in the model, an orphan disease was defined as a condition for which at least one orphan medicine is licensed; an orphan disease came into existence in the model during the year in which the first medicine designated for that condition was licensed. By using the orphan disease, rather than the orphan drug, as the primary unit, the model better accounts for the fact that many new orphan drugs are indicated for conditions in which an approved orphan drug is already available. The cost impact of having multiple drugs available in one disease (such as in the treatment of pulmonary arterial hypertension) is accounted for in the model by estimating the average penetration rate from diseases with both single and multiple drugs.

For years 2001 to 2010, the number of orphan diseases in existence in Europe was determined by reviewing the European Commission registry of orphan medicines with marketing approval, and identifying the number of discrete indications [4].

For years 2011 - 2020, the number of new orphan diseases was forecast based upon the following assumptions:

*Orphan drug designations rate *after 2010 will grow at 10% per annum between 2011 and 2020, reflecting the average growth rate in designations between 2001 and 2010.

*Proportion of drugs with novel indications *(as opposed to drugs being designated for conditions in which at least one licensed orphan medicine is already approved) was assumed to be the same as for all orphan drug designations between 2000 and 2010 - 43% of designated drugs are for indications in which no other orphan drug is yet licensed.

*'Success rate' *(the proportion of designated orphan drugs that are given marketing approval) remained constant at 10.9% of all designations - the level observed over the last decade. Explanation for this seemingly low success rate is provided in the Discussion section.

*Lag time between designation and approval*. Based upon an analysis of the European Commission registry of orphan medicines approved for use in Europe between 2000 and 2010, the average lag time between being granted orphan drug designation and achieving marketing authorisation was 4 years. Drugs given orphan drug designation before 2003 that had not received marketing approval by the end of 2010 were assumed to be unsuccessful in reaching the market.

### Average annual orphan drug cost per orphan disease

The average orphan disease drug cost was estimated for each year of the lifecycle of an average orphan disease, starting from the year in which the first orphan medicine was licensed for use in that indication. This reflects the fact that the cost of orphan medicines for an orphan disease will change over time: growing from the year in which the first drug is launched, as more patients gain access to treatment, until the point that the first drug becomes generic, at which point the total cost of orphan drug treatment in that disease falls.

The orphan drug cost for an average orphan disease was estimated based upon data on all currently licensed orphan medicines in Europe. The drug cost was estimated for each existing orphan disease (for which a designated orphan medicine was approved) and then an average was taken across all the diseases.

Individual disease drug costs were calculated using the following information:

• Annual orphan drug costs for each disease were calculated based on the information in the manufacturer's Summary of Product Characteristics (SPC). Drug dosages reflected the average dose for an adult, unless the drug was specifically indicated for use in children. The duration of treatment (chronic, long-term, short-term, cyclical or episodic) was obtained from the SPC. Where treatment was administered as an injected or infused drug, the nearest full vial size was used.

• Prevalence estimates from published data [14] and EMA regulatory filings were used to quantify the potential patient population for each disease for every country included in the analysis.

• Availability of orphan medicines across the countries included in the analysis was estimated based upon a survey of 10 European countries that found that, of patients with diseases for which an orphan drug was licensed, 69% on average had access to the medicine [15]. This estimate is commensurate with reports that show that on average only approximately two-thirds of licensed orphan medicines are available for patients to access across Europe [15-17]. The percentage of the total prevalent patient population likely to receive treatment in each year after the introduction of the first orphan medicine was estimated based upon historical sales data from a sample of orphan drugs approved in Europe since 2001. Using estimated prevalence as the denominator, the average uptake rate was seen to be 22% of the prevalent population in 2010. It should be noted that this factor is based upon the proportion of patients estimated to have the condition based upon prevalence estimates, rather than those diagnosed individuals with clinically significant disease in whom treatment with an orphan drug would be indicated. Therefore the true 'uptake rate' amongst patients for whom treatment is appropriate is likely to be much higher than this. However, there is insufficient published data to estimate the smaller patient population indicated for each orphan treatment, and as such total prevalence data was used, with the consequence that the uptake rate figures appears artificially low. This does not affect the validity of the model, as the same prevalence-based denominator was used for both the retrospective analysis of historical data and the forecast of future orphan cost growth.

To account for the effect of loss of intellectual property and marketing exclusivity on orphan medicines, drug costs were assumed to fall in price by 25% from the 10^th ^year after the introduction of the first orphan medicine in a disease area. This is the average reduction in price for medicines at the point of loss of patent observed between 2000-2007 in the European Commission Pharmaceutical Sector Inquiry Report [18]. The extent to which this is analogous to the orphan drug market is explored in the Discussion section.

The year-on-year cost curve for the average orphan disease was mapped onto the forecast incidence of new orphan diseases to predict the total budget impact at any time point. For each year, this total cost was divided by the estimated total pharmaceutical expenditure of the Eurozone countries plus UK that was obtained from the pharmaceutical industry trade body [19]. Estimates of future total pharmaceutical market value (ex-factory) were made based upon the growth rates seen over the previous 5 years.

One-way sensitivity analysis was performed on a range of variables to establish how robust the budget impact estimates were to changes in key parameters and assumptions.

## Results

### Predicted number of orphan diseases

**Figure 1:**
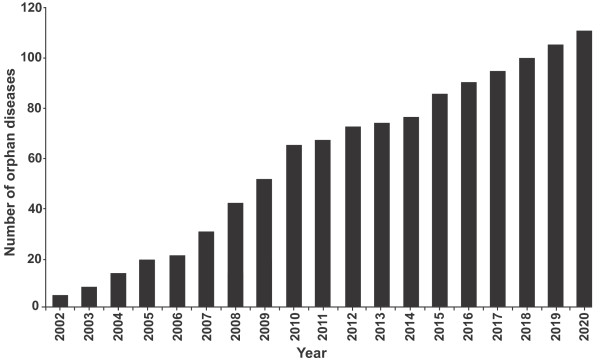
**Cumulative orphan diseases: observed (2002 - 2010) and forecast (2011 - 2020)**.

Figure 1 shows the cumulative number of orphan diseases (defined in this study as a disease in which at least one designated orphan drug is licensed) observed between 2002 and 2010, and the forecast number of cumulative orphan diseases between 2011 and 2020.

The trend is towards a steady increase in the cumulative number of diseases for which an orphan drug is designated, averaging just over 5 new diseases per year over the 20 years of the model. The rate of growth in diseases was more rapid between 2002 - 2010 than 2011 - 2014, reflecting the acceleration in orphan drug designations between 2001 and 2005 and the slowing growth of designations between 2006 - 2010 (the average lag between designations and approvals being approximately 4 years). From 2015 forth, the additional increase in orphan diseases is forecast in line with a designation rate between 2010 - 2015 that is premised upon designations growing at the average rate over the period 2001 - 2010 (approximately 10% per year).

### Average cost per orphan disease per year

**Figure 2:**
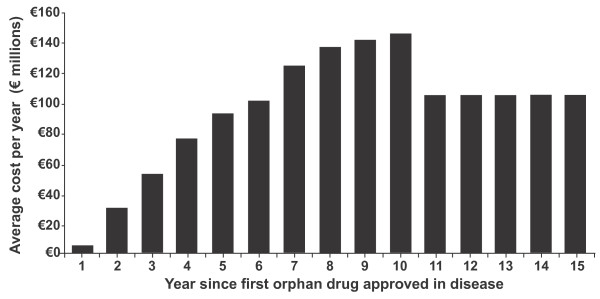
**Average annual drug cost per orphan disease over the indication lifecycle, for the Eurozone + UK**.

The annual per patient cost of existing orphan drugs was seen to vary between €1,251 and €407,631, with the median cost being €32,242. When combined with the expected drug penetration rate within the prevalent patient populations, the average orphan disease drug cost across the Eurozone and UK was predicted to rise from €5 m in the year after the first orphan drug was approved, to €143 m in Year 10, before falling and steadying-off at approximately €110 m per year (Figure 2).

### Predicted budget impact of orphan drugs in Eurozone and UK

The ex-factory value of the total pharmaceutical market for the countries included in this analysis was estimated to be €140 bn in 2010, based upon data from the European Federation of Pharmaceutical Industries and Associations (EFPIA) [19]. The growth of the total market over the next decade was estimated to follow the same trend identified by EFPIA over the last 5 years - annual growth of 6.6% [20]. Accordingly, the total pharmaceutical market in the Eurozone and UK is expected to grow to €265 bn by 2020. Despite recent price cutting by some European countries, the fundamental demographic trends in Europe are likely to support continuing drug market growth within this range [21].

**Figure 3:**
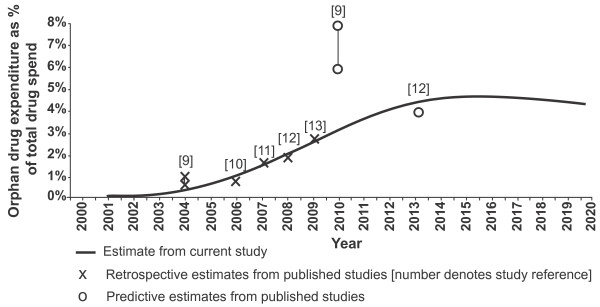
**Budget impact of orphan drugs as percentage of total pharmaceutical spend (2002 - 2020)**.

According to the results of the model, the share of the total European pharmaceutical market represented by orphan drugs is predicted to increase from 3.3% in 2010 to a peak of 4.6% in 2016 before steadying-off at a level between 4% and 5% until 2020 (Figure 3). The flattening of growth in the budget impact from 2016 onwards does not mean that the total cost of orphan drugs is expected to stop growing, but rather that the growth of orphan drug expenditure is unlikely to be significantly greater than that of the total pharmaceutical market during this period.

Within the total budget impact, 40% of the diseases for which orphan medicines were licensed were for oncological and haematological conditions, yet these diseases accounted for 57% of the total costs in 2010. While the average annual cost for oncology treatments was significantly lower than the average annual cost of non-oncology treatments (€33,919 and €86,145 respectively), the prevalence of these conditions is on average higher. For example, the prevalence for Gaucher's Disease is 2 per 100,000 population in contrast to a prevalence of 32 per 100,000 for B-cell chronic lymphocytic leukemia.

Deterministic sensitivity analysis demonstrated that the forecast was not unduly sensitive to changes in any key parameter (Table 1). In all scenarios the peak-year budget impact fell within a range of 3% - 6.6%. Changes to the predicted rate of total pharmaceutical growth from 6.6% (average over last 5 years) to 3% (the lower end of current industry analyst projections [21] saw the peak year budget impact increasing to 5.9%. When the impact of generic entry was excluded from the analysis, the peak-year budget impact increased to 5.1%. The greatest budget impact estimate was associated with increasing the 'strike-rate' parameter (percentage of designated orphan drugs that go on to receive marketing approval) to 20% - a rate that is significantly higher than that observed in either the EU or US since orphan legislation was introduced.

**Table 1:** Results from one-way sensitivity analysis on key model parameters.

Parameter		Value		Peak year budget impact as % of total pharmaceutical spend (year)
1	Lag between orphan drug designation and marketing approval	Base	4 yr	4.6% (2016)
		Best	6 yr	4.2% (2016)
		Worst	2 yr	4.9% (2015)

2	Eurozone + UK pharmaceutical market value in 2010 (ex-factory)	Base	€140 bn	4.6% (2016)
		Best	€170 bn	3.8% (2016)
		Worst	€110 bn	5.8% (2016)

3	Total pharmaceutical market growth per year	Base	6.6%	4.6% (2016)
		Best	10%	4.0% (2014)
		Worst	3%	5.9% (2020)

4	% of products with orphan designation that achieve market authorisation	Base	10.92%	4.6% (2016)
		Best	7%	4.2% (2015)
		Worst	20%	6.6% (2016)

5	Growth in new designations per year after 2010	Base	10%	4.6% (2016)
		Best	5%	4.6% (2016)
		Worst	50%	4.7% (2020)

7	Average cost of orphan disease per year (Yr 1 - Yr 10)	Base	As per Figure 2	4.6% (2016)
		Best	-25% p.a	3.4% (2016)
		Worst	+25% p.a	5.7% (2016)

8	Drop in drug prices at the point that drugs lose patent protection or marketing exclusivity	Base	25%	4.6% (2016)
		Best	50%	4.2% (2014)
		Worst	0%	5.1% (2019)

('Best' and 'worst' cases are from the perspective of funders of orphan medicines, with 'best' representing the lower budget impact and 'worst' the higher)

## Discussion

The findings from this analysis demonstrate how the budget impact of orphan drugs in Europe has grown steadily over the 10 years since the introduction of the orphan drug regulation in 2000, driven primarily by the approval of new drugs for diseases in which no treatments were previously licensed. The budget impact is estimated to have grown from 0% in 2000 to 3.3% by 2010. From 2010 to 2016 this growth is predicted to continue, however the rate of growth is likely to slow and will reach a plateau of approximately 4.6% of total pharmaceutical market expenditure by 2016. However, although the budget impact, as a percentage of total pharmaceutical expenditure, is not expected to grow beyond 2016, the absolute expenditure on orphan medicines will increase year-on-year, but no faster than the growth in the greater European pharmaceutical market.

What explains the steadying growth of orphan medicine budget impact over the next decade, forecast in this analysis? One factor is the loss of marketing exclusivity and patent protection for orphan drugs that were introduced shortly after the passing of legislation in 2000. For example, major orphan medicines such as imatinib and bosentan will both lose marketing exclusivity and patent protection by 2015 [22,23].

**Figure 4:**
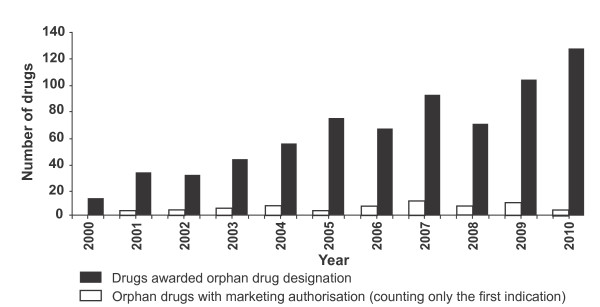
**Orphan drug designations and approvals in Europe (2000 - 2010)**[4].

A second factor is the low 'success rate' for drugs that have been granted orphan designation. Over the first 10 years since the introduction of European orphan drug legislation, the success rate for approvals per designation has averaged 10.9%. While designation rates have grown steadily over this period, approvals of new treatments have fluctuated, and in fact fallen from 13 per year in 2007 to 4 in 2010, as shown in Figure 4.

A third reason for the steadying growth of orphan drug costs is the diminishing penetration rate of new drugs into prevalent patient populations in the later years after a drug is first licensed. Of the whole prevalent population with an orphan disease, only a certain percentage will be diagnosed and be appropriate for treatment [24]. Those patients most in need of treatment will be identified quickly, but the marginal uptake rate diminishes over time. Access issues - related to pricing and reimbursement restrictions - will also continue to constrain the patient uptake of new and existing orphan drugs. A patient access survey conducted by EURORDIS found that some orphan drugs that have been available on the market for many years are still not becoming more widely available over the course of time [15]. Even excluding the drop in costs associated with loss of marketing exclusivity, this slowing in uptake in the latter part of the drug-disease lifecycle dampens the overall growth in orphan drug expenditure.

Combined, these factors mean that the fast cost growth seen in the past decade is unlikely to continue in the forthcoming one, with designations and approvals of new diseases likely to be at a 'replacement level' that maintains growth in orphan expenditure at a trajectory that is similar to that of the greater pharmaceutical market.

This analysis has sought to forecast future events - a process that is inherently subject to uncertainty, assumption and potential bias. The principal form of structural uncertainty in the model comes from the decision to use orphan diseases as the primary unit of measurement rather than individual orphan drugs. By structuring the model in this fashion, the cost associated with multiple therapies for a single disease has been incorporated via the estimated 'penetration rate' of potential patients receiving treatment in each disease. This rate was estimated from existing orphan diseases where both single and multiple drugs were available.

Parameter uncertainty in the model reflects the nature of the data sources used. The European Commission register of orphan designations and approvals is a robust source for identifying trends in these areas, yet data on prevalence in individual diseases is relatively weak, and comparing drug prices across European markets introduces significant complexity. Ex-factory orphan drug prices were used in the model, but this may not reflect the true price paid, excluding as it does discounts, rebates and risk-sharing schemes.

Equally importantly, the potential price erosion when older orphan drugs lose their patents and marketing exclusivity is difficult to accurately predict. Although there has been insufficient time to assess this change for orphan medicines in Europe, in the US, where orphan legislation was introduced in 1983, the impact of loss of marketing exclusivity appears to have resulted in significant changes in price upon the introduction of generic competitors. An analysis of a sample of 12 drugs that received orphan drug approval in the US between 1990 and 2000 suggested that on loss of marketing exclusivity, generic prices were on average 50% lower than the original drug, with a range of price reductions from 14% to 95%. Adopting a 25% fall in price is therefore considered conservative.

In light of the economic pressure on healthcare and social security budgets, there may also be uncertainty as to whether the pharmaceutical market growth rate will continue to be sustained at the historic level of 6.6% per annum. However, more recent reports on the pharmaceutical industry expect the market to grow 3% - 6% annually through 2014 [21]. Based on the conservative estimate of a 3% annual growth in the total market, the orphan drug budget impact would be 5.9% in 2020 (Table 1).

The 'success rate' of obtaining marketing approval for drugs that have been granted orphan designation was also seen to be an important variable in sensitivity analysis. Over the first 10 years since the introduction of the European orphan drug legislation, the success rate for approvals per designation has averaged 10.9%. This rate is comparable with the 15.9% success rate observed in the US over the 28 years since orphan legislation was introduced in 1983. This relatively low success rate reflects the broader attrition rate seen in all areas of pharmaceutical research and development (R&D) and is less an indication of failure, than a reflection of the importance of orphan legislation R&D incentives to drug manufacturers. Companies are incentivised to register a treatment for orphan drug designation even if the probability of success is low (as is often the case in drug research). Clinical trial data is not a prerequisite for orphan drug designation; pre-clinical evidence of effectiveness is sufficient. As manufacturers become more aware of orphan drug R&D incentives, more drugs are being designated prior to proof-of-concept, with subsequently lower likelihood of drugs succeeding to marketing authorisation.

The 'uptake rate' used in this analysis is also subject to uncertainty. The potential patient population for any one disease - the denominator in the uptake rate - is derived from published prevalence data, but such data only reflects the theoretical total population with the disease. It is therefore not a true reflection of the uptake rate within the patient population who are appropriate for treatment. For example, anagrelide is indicated for essential thrombocythemia. However, the European license restricts its use to a patient population who are intolerant to the established first-line treatment with hydroxycarbamide - a much smaller sub-population [25,26]. Similarly, in Gaucher's disease prevalence estimates indicate that 5000 patients should exist in a country such as Germany, yet as of 2010 only 250 (5%) patients were receiving treatment with enzyme replacement therapy there, despite a product having been available since 1994 [24].

Despite this uncertainty, sensitivity analysis has shown the model to be robust to changes in key parameter values and there is relatively little variation in the expected budget impact of orphan medicines over the next 10 years, with all results from sensitivity analysis falling below 6.6% of total pharmaceutical market sales.

In addition, the estimates from the model are supported by other related literature in this field. Figure 3 overlays the published estimates from other European researchers on the results from the study discussed here. As can be seen, the historical estimates from the model (2001 - 2010) fit closely with those produced from other sources. The future estimates of budget impact in this analysis also aligns well with the forecast for 2013 budget impact from the Belgian Federal Centre for Healthcare, which also expected the total budget impact to be approximately 4% at that time. The only existing analysis that is incongruous with our findings was that conducted on behalf of the European Commission, which predicted that the orphan drug budget impact would be between 6% and 8% by 2010. However this estimate was made in 2004, based upon the small body of historical data that was available at that time.

Despite the similarity in findings, the analysis reported here differs in methodology from those studies reported previously. It is the first study to attempt to estimate the budget impact for the region, rather than for specific markets, which improves the relevance of the results for policy makers at a European level, but possibly at the price of lower sensitivity to local variations in orphan medicine provision. It is also the first study to forecast forward over a decade, whereas previous studies have been retrospective [10,11,13] or have forecast forward over relatively short periods [9,12]. This more ambitious scope could create greater uncertainty in the analysis, but at the same time there is more retrospective data upon which this analysis is based compared with earlier studies.

Given the importance of this topic, and the uncertainties in this analysis, further research is required to inform orphan drug policy moving forward. As more data becomes available on growth of orphan expenditure, future extrapolations will have greater certainty and less variance. It will also be valuable to investigate the likely cost of orphan drugs on a market-by-market basis, as despite the common assumptions used in this study, there are likely to be difference between countries. Further investigation into the composition of orphan drug cost may also be valuable.

## Conclusions

Despite the discussed limitations and the uncertainty within some parameters, this forecast provides a useful insight into the likely budget impact of orphan diseases in Europe in future years, and may aid healthcare policy makers when planning for the funding of such medicines. Although orphan drug regulation has led to an increase in the budget impact of orphan drugs, the cost, as a proportion of total pharmaceutical expenditure, is likely to plateau between 4% - 5%. Fears of unsustainable cost escalation should not be used as rationale to review the orphan drug regulation.

## Competing interests

This study was funded by Celgene Corporation, a biopharmaceutical company that discovers, develops and manufactures medicines, including some for rare diseases. CS and AH also provide paid consultancy services to other pharmaceutical companies, some of whom manufacture medicines for rare disease. TM is an employee of Celgene.

## Authors' contributions

CS, TM and AH jointly determined the analytical structure of the analysis. CS and AH constructed and populated the model and ran analyses. TM provided data for inclusion in the model. CS, TM and AH all participated in interpreting and reporting the study findings. All authors read and approved the final manuscript.
